# Using a mHealth tutorial application to change knowledge and attitude of frontline health workers to Ebola virus disease in Nigeria: a before-and-after study

**DOI:** 10.1186/s12960-016-0100-4

**Published:** 2016-02-12

**Authors:** Akaninyene Otu, Bassey Ebenso, Okey Okuzu, Egbe Osifo-Dawodu

**Affiliations:** Department of Internal Medicine, College of Medical Sciences, University of Calabar, P.M.B. 11115, Calabar, Cross River State Nigeria; Nuffield Centre for International Health and Development, Leeds Institute for Health Sciences, University of Leeds, Leeds, UK; Instrat Global Health Solutions, New Jersey, USA; Instrat Global Health Solutions, Abuja, Nigeria; Anadach Group, Azusa, CA USA

**Keywords:** Ebola virus disease, Improving knowledge and attitude, mHealth, Frontline health workers, Nigeria

## Abstract

**Background:**

The Ebola epidemic exposed the weak state of health systems in West Africa and their devastating effect on frontline health workers and the health of populations. Fortunately, recent reviews of mobile technology demonstrate that mHealth innovations can help alleviate some health system constraints such as balancing multiple priorities, lack of appropriate tools to provide services and collect data, and limited access to training in health fields such as mother and child health, HIV/AIDS and sexual and reproductive health. However, there is little empirical evidence of mHealth improving health system functions during the Ebola epidemic in West Africa.

**Methods:**

We conducted quantitative cross-sectional surveys in 14 health facilities in Ondo State, Nigeria, to assess the effect of using a tablet computer tutorial application for changing the knowledge and attitude of health workers regarding Ebola virus disease.

**Results:**

Of 203 participants who completed pre- and post-intervention surveys, 185 people (or 91%) were female, 94 participants (or 46.3%) were community health officers, 26 people (13 %) were nurses/midwives, 8 people (or 4%) were laboratory scientists and 75 people (37%) belonged to a group called others. Regarding knowledge of Ebola: 178 participants (or 87.7%) had foreknowledge of Ebola before the study. Further analysis showed an 11% improvement in average knowledge levels between pre- and post-intervention scores with statistically significant differences (*P* < 0.05) recorded for questions concerning the transmission of the Ebola virus among humans, common symptoms of Ebola fever and whether Ebola fever was preventable. Additionally, there was reinforcement of positive attitudes of avoiding the following: contact with Ebola patients, eating bush meat and risky burial practices as indicated by increases between pre- and post-intervention scores from 83 to 92%, 57 to 64% and 67 to 79%, respectively. Moreover, more participants (from 95 to 97%) reported a willingness to practice frequent hand washing and disinfecting surfaces and equipment following the intervention, and more health workers were willing (from 94 to 97%) to use personal protective equipment to prevent the transmission of Ebola.

**Conclusions:**

The modest improvements in knowledge and reported attitudinal change toward Ebola virus disease suggests mHealth tutorial applications could hold promise for training health workers and building resilient health systems to respond to epidemics in West Africa.

## Background

The Ebola virus disease (EVD) outbreak in West Africa commenced in a remote area of southern Guinea in December 2013 while the first cases were reported in March 2014. Unlike previous outbreaks in East and Equatorial Africa [1] that were brought under control fairly swiftly, the West African outbreak is considered the worst in history due to an almost unimpeded spread from Guinea to Sierra Leone, Senegal, Liberia, Mali and Nigeria [2, 3]. The growing concern that followed its spread to other countries and the cumulative number of deaths led to the declaration of EVD as a Public Health Emergency of International Concern by the World Health Organization (WHO) in August 2014 [2]. EVD has a case fatality rate of 60–90%, and there is still no available drug or vaccine [3] for the disease [4].

The initial spread of EVD in West Africa was fuelled by socio-cultural factors and a collective failure to ensure availability of adequate health staff, basic healthcare resources and systems in the most affected countries [5, 6]. Subsequent patterns of spread revealed that health services in affected countries were overstretched by the epidemic [7] and that frontline health workers (FHWs) were at increased risk of contracting EVD by coming in contact with body fluids of infected patients. Many health workers who dealt with the outbreak were neither provided with protective clothing nor equipment to reduce their risk of infection when caring for patients and/or the deceased. In the USA and Spain where personal protective equipment was in abundant supply, FHWs were put at risk by inadequate training on how to safely provide medical care to persons with EVD. This situation led to an unprecedented number of infections and deaths among FHWs, thus depleting a vital asset during the control of the outbreak. By 12 April 2015, about 868 (or 3.4%) cases of Ebola reported globally were among health workers, 503 (or 2%) of who died from the disease [2, 6].

Understandably, the moral, physical and psychological consequences of the epidemic dampened the enthusiasm of FHWs [8] to provide essential services in affected countries. The hesitancy of FHWs to put themselves in harm’s way placed an even greater strain on fragile health systems. To counteract this situation, the presidents of affected countries and the Director-General of the WHO launched a joint $100 million emergency response to control the epidemic, including through providing adequate protective clothing, training and support for anyone who was in contact with Ebola patients [7]. Integral to the emergency plan were collaborations between national governments and mobile network providers to harness the high penetration of mobile phone subscription in Guinea (63/100 people), Liberia (60/100 people) and Sierra Leone (44/100 people) to provide low-cost, high-impact mobile health (mHealth) solutions [9] for mapping outbreaks, providing education interventions to support behaviour change [10] and providing reference and training materials that FHWs can download onto their phones [8, 11]. In many ways, the successful utilization of advanced wireless technologies to fight the Ebola outbreak in Nigeria drove the increased focus on mobile phones elsewhere in the region. These partnerships aimed to tap into phenomenal growths in information and communication technology (ICT) in low- and middle-income countries (LMICs), to improve health and healthcare services in countries affected by Ebola.

The use of mHealth technology refers to the incorporation of wireless devices such as mobile phones, tablets, personal diagnostic assistants (PDAs), patient-monitoring devices and other wireless devices into medical and public health practice to improve health or ensure healthcare [9, 11–13]. Recent reviews show the explosive innovation in technology has inspired a proliferation of mHealth pilot initiatives, many of which have demonstrated that mHealth can alleviate specific health system barriers such as balancing multiple priorities, lack of appropriate tools to provide services and collect data, and limited access to training and supervision [14, 15].

Despite the utility of mHealth for strengthening health systems in a range of health fields including mother and child health (MCH), management of diabetes and HIV treatment [11], there is little empirical evidence of the effectiveness of mHealth in improving health system functions during the West African Ebola epidemic. This paper therefore aimed to assess the effect of using a tablet computer application to deliver an education intervention to change frontline health workers’ EVD-related knowledge and attitude in Nigeria. We sought to answer the following questions: (a) are tablet computer applications effective for streaming EVD-related tutorial to FHWs, in the context of an epidemic, and (b) what is the effect of the education intervention (tutorial) on FHWs’ knowledge about and attitude toward EVD? The hypotheses were that (i) EVD-related information can be delivered at a distance from tablet computers to FHWs in the context of an epidemic and (ii) tablet-delivered education intervention could positively change FHWs’ biomedical knowledge of EVD.

### Conceptual clarification of terms

#### mHealth interventions

As already mentioned in the preceding paragraphs, mHealth uses wireless devices to support medical and public health practice to improve health or healthcare [16]. mHealth interventions may include, e.g. using mobile short text messages (SMS) to stimulate the uptake of service and patient adherence to medication as well as to educate caregivers and ensure provider compliance with clinical protocols [17].

#### Education interventions

The aim is to provide intervention participants (patients, general public or health providers) with disease-specific information [18] to increase awareness and knowledge of symptoms, causes and consequences of diseases in order to improve disease control and promote healthcare-seeking behaviour in populations [19, 20]. Some scholars, however, claim that education interventions can also change beliefs and attitudes toward disease conditions [21]. Unlike mHealth interventions which primarily use mobile/wireless devices to support improvements in health or healthcare, education interventions, on the other hand, can be delivered through a range of strategies including the following: traditional face-to-face lectures, videos and films, internet-based approaches and mobile phone and tablet technology to enable training of participants [22].

This paper reports an education intervention that used tablet computers as a medium to facilitate the training of frontline health workers. The intervention was a component of the ‘Front Line health worker Education and disease Management (FLEM) project’, developed by *Instrat* and *Anadach* and implemented on the Vecna Cares CliniPAK system under a Qualcomm® Wireless Reach™ grant. The FLEM project was implemented in collaboration with the Ondo State Government in Nigeria with the objective of improving emergency preparedness of the health system to contain the Ebola outbreak.

## Methods

### Design

The study consisted of quantitative cross-sectional surveys in selected health facilities, with pre- and post-intervention knowledge, attitude and practice (KAP) measurements of health workers.

### Setting

This research is set in Ondo State, western Nigeria. Ondo State has a population of 3.9 million and is made up of 18 local government areas (LGAs) with Akure as its capital city [23–25]. The state has about 800 primary health facilities, 18 general hospitals and 6 tertiary health facilities [26]. Primary health facilities are managed by LGAs and funded through statutory allocations from the state government. The doctor/patient ratio in the state is 1:14 000 as against 1:5000 recommended by the WHO. A recent study shows that human resources for health (HRH) employed by the state government include the following: 148 medical doctors, 908 nurses, 137 medical laboratory technologists and scientists, 185 Community Health Officers (CHOs) and 1152 community health extension workers (CHEWs) [27, 28]. Evidence also shows an inequitable distribution of qualified HRH, especially doctors and nurses, with higher ratios per population in the urban compared to rural areas [25]. Regarding health indicators, Ondo State has a maternal mortality rate of 371/100 000 live births and an infant mortality rate of 68/1000 live births, considered by the World Bank in June 2009 as the worst health indices in South West Nigeria [28]. This catalysed the ministry of health (MOH) to provide health services free of cost to pregnant women and children aged <5 years as a strategy for improving access to health. Additionally, the government supplied health facilities with tablet computers loaded with an electronic health record system (a proprietary system—*CliniPAK* application) to improve data collection and ultimately health outcomes.

### Participants and facilities

Eligible participants were doctors, nurses, midwives, laboratory technicians, CHOs and CHEWs drawn from 14 health facilities across Ondo State purposively selected because the facilities were already supplied with tablet computers loaded with an electronic health record system. We approached the heads of the 14 facilities, explained the objectives of the study and invited FHWs from the facilities to participate in the study. All 14 facilities are equipped with and use Vecna Cares’ CliniPAK health data capture system for the daily documentation of MCH care delivered in the clinics. The application leverages the CommCare Open Data Kit platform to provide an easy-to-use electronic health record system front-end interface that runs on tablet computers.

### Educational intervention

The evaluation of the front line health worker education and disease management (FLEM) application intervention consisted of (i) pilot testing a tablet computer tutorial application for improving diagnostic and management responses to EVD and (ii) conducting before and after surveys to assess changes in the KAP of FHWs regarding EVD. The electronic tutorial application was designed by a multidisciplinary group of ICT and infectious diseases experts and health system researchers to extend and enhance existing CliniPAK electronic health information systems to disseminate critical information to FHWs in real time via tablet computers. The application is unique in that it requires minimal instruction for use after download and installation. Following the development of the FLEM application, an orientation programme was organized for FHWs of the facilities during which potential participants were trained to use tablet computers and the CliniPAK electronic application. As part of the orientation, the objectives of the study were explained to FHWs after which they were invited to participate in the study. Participants were subsequently provided with instructions on completing a pre-tutorial survey, reviewing the electronic Ebola tutorial and completing a post-tutorial survey.

Participants were assured of absolute confidentiality and the right to either accept or refuse to participate without consequences. However, due to delays in receiving ethical clearance for the study and the time required for pre-testing the tutorial application, the baseline data collection commenced on 3rd October 2014 during the epidemic while the post-intervention data collection was completed on 25th February 2015 after Nigeria was declared Ebola-free. Ethical clearance for the study (number G.8061/99) was obtained from Ondo State Ethical Review Committee.

The study was implemented via the following steps:

Step 1: All participants completed a consent form electronically.

Step 2: Participants inputted demographic information into the FLEM application.

Step 3: Participants were invited to complete a pre-tutorial knowledge and attitude assessment (pre-KAA), to establish a baseline of health workers’ knowledge of and attitudes toward EVD. The assessments contained objective knowledge questions as well as subjective attitudes and perception questions.

Step 4: Ebola awareness tutorial (EAT) was launched on all tablet computers deployed in 14 healthcare facilities in Ondo State. This educational intervention/course comprised essential information on EVD namely the source, incubation period, clinical features and route of spread. Other areas covered included transmission, diagnosis of EVD, clinical management and prevention of Ebola transmission in healthcare settings. Health workers that completed the pre-KAA were required to view the EAT at their convenience over a 2-week period. The 2-week period was chosen to keep the duration of the education intervention short in the context of the FLEM project objective of improving emergency preparedness of the health system to contain the Ebola outbreak. Participants were allowed multiple views of the EAT.

Step 5: A post-tutorial knowledge and attitude assessment (post-KAA) was launched to establish the effectiveness of the training on the health workers. The questions asked on the post-KAA were exactly the same as those asked during the pre-KAA to facilitate comparison of performance. All participants who completed the post-KAA were expected to have done the pre-KAA and viewed the EAT on at least one occasion. Tablet computers had prepaid annual subscriptions to mobile networks to circumvent the issue of accessing mobile phone networks while health workers were using the tablets. The responses for the pre- and post-KAA were stored electronically for each participant and subsequently analysed.

### Analysis

The pre- and post-KAA data and the logs on all frontline workers who viewed the EAT were downloaded from the CommCare survey data repository database to a Microsoft Excel Package (version 14.0.7145.5000 (32-bit). Two hundred and eighty-two frontline workers completed the pre-KAA survey, while 214 workers completed the post-tutorial survey. We excluded data for 11 workers who did not view the tutorial but completed the post-KAA survey. The study analysis was thus conducted on data for 203 workers who completed the pre- and post-tutorial surveys and viewed the tutorial.

The knowledge part of the study contained nine objective questions. We assigned scores of 1 for each correct answer and 0 for wrong answers that frontline workers provided. We counted the number of respective responses to arrive at the number of FHWs that selected each outcome and computed a percentage of aggregate counts for each outcome relative to the total and compared the pre- and post-tutorial scores to arrive at the difference in health worker knowledge of the Ebola virus disease. A paired sample *Z* test was done on the means of the pre- and post-tutorial scores for knowledge questions, and the significance level was set at *P* < 0.05.

The behavioural and attitudinal part of the study contained seven questions with five possible choices: Strongly Agree, Agree, Not Sure, Disagree, and Strongly Disagree. To streamline interpretations of health worker responses, we combined ‘Strongly Agree and Agree’ to derive ‘Agree’ and ‘Strongly Disagree’ and ‘Disagree’ to derive ‘Disagree’. We thus had three possible outcomes: Agree, Not Sure or Disagree. We counted the number of respective responses to arrive at the number of FHWs that selected each outcome from each health facility. We then took simple averages of the response counts across all the facilities to arrive at the average score for each of the seven questions. Finally, we computed the percentage of aggregate counts for each outcome relative to the total and compared the pre- and post-tutorial scores to arrive at the difference in health worker behaviours and attitudes toward the Ebola virus disease.

## Results

Only 2 of 14 participating facilities were tertiary-level facilities (see Table 1 for Akure and Ondo MCH hospitals). The remaining 12 facilities consisted of 6 secondary-level comprehensive health centres and 6 primary-level basic health centres, selected to represent the three senatorial districts of Ondo State (Ondo North, Ondo Central and Ondo South). Two hundred and three FHWs (or 72%) of the recruited 282 participants completed the pre-KAA, EAT and post-KAA surveys. Fifty-five of the 203 health workers (or 27%) were from Akure MCH located in the State capital while just two health workers (or 1%) from Odo-aiye health centre (Table 1). The majority of participants were female (185 people; 91%). There were 94 (46.3%) community health officers/primary healthcare workers (PHC), 26 (12.8%) nurses/midwives, 8 (3.9%) laboratory scientists and 75 (37%) in the subgroup of ‘others’ which comprised of auxiliary nurses, pharmacy technicians and health record staff.

**Table 1:** Number of participants in the study by Health Facility in Ondo State

Name of facility	No. of participants drawn from facility	Percent
Akure MCH	55	27
Ondo MCH	27	13
Oke-Igbo	27	13
Ore	28	14
Arakale	18	9
Odode	18	9
Molete	12	6
Ikare	7	3
Uso	5	2
Oka	4	2
Odo-aiye	2	1
Total	203	100

*MCH* mother and child health

One hundred and seventy-eight participants (or 87.7% of FHWs) had heard about EVD prior to the study while 25 (12.3%) had not. Most health workers (116; 57.1%) learnt about Ebola mainly from the radio and television (see Table 2).

**Table 2:** Source of knowledge of EVD among participants prior to intervention

Source of knowledge	Number of participants (*n* = 203)	Percent
Friends or family	7	3.5
Internet and social media sites such as Google, Facebook and Twitter	20	9.9
Newspapers	8	3.9
Radio and television	116	57.1
Work	50	24.6
Other	2	1

*EVD* Ebola virus disease

### Knowledge of EVD

Four of the nine knowledge questions had a single possible correct answer that would score 1 point if that answer was correct or 0, if wrong. The other five questions were designed to have more than one answer possible. We prorated the scoring for each correct answer to the five questions such that a FHW who correctly selected one out of two correct answers was awarded a score of 5.55 for that question.

Analysis of the knowledge assessment showed an increased trend after pre-KAA results when compared with post-KAA results. However, the question that assessed knowledge on the natural host of EVD showed a decrease when post-KAA results were compared with pre-KAA results (Table 3). Also, respondents tended to regard EVD as a less deadly disease following exposure to the educational materials.

**Table 3:** Pre- and post-tutorial scores for correct responses to knowledge questions

Question no.	Description of question	Pre-tutorial % FHWs with correct responses	Post-tutorial % FHWs with correct responses	Delta score (% of difference/pre-EAT score)	*P* value from *Z* test
Question: 1	As far as you know, what is the natural causative agent of Ebola fever?	100.0	99	−1.5	0.08
Question: 2	As far as you know, what is the natural host of Ebola fever?	8.9	7.4	−17	0.5
Question: 3	As far as you know, Ebola fever can be transmitted from human to human by (more than one answer is possible)	47.3	63.5	34	0.001^a^
Question: 4	Common symptoms of Ebola fever include (more than one answer is possible)	41.4	64.0	55	0.001^a^
Question: 5	As far as you know, can Ebola be treated?	38.4	44.8	17	0.19
Question: 6	Ebola fever is treated with	93.6	97.0	4	0.10
Question: 7	As far as you know, is Ebola fever preventable?	44.8	60.6	35	0.001^a^
Question: 8	Ebola fever may be prevented by (more than one answer is possible)	99.5	99.5	0	1
Question: 9	Do you think Ebola fever is a serious disease that can lead to death	80.8	78.3	−3	0.54
Average knowledge (%)		61.6	68.2	11	

^a^Statistically significant

*EAT* Ebola awareness tutorial, *FHW* frontline health worker

When disaggregated by professional category, the average pre-EAT knowledge scores of EVD were highest among nurses/midwives (70.9) and lowest among the subgroup of ‘other’ health workers (55.6). However, the general upward trend in average between pre- and post-EAT scores was reflected among all professional categories with the greatest change in average score of 10.1 being recorded among PHC workers (Table 4).

**Table 4:** Pre- and post-EAT average scores by professional category

Professional category	Pre-EAT score average	Post-EAT score average	Difference between pre- and post-EAT score	Delta score (% of difference/pre-EAT score)
PHC worker	62.5	72.6	10.1	16
Nurse/midwife	70.9	72.2	1.3	2
Lab scientist	66.7	73.6	.9	10
Other	55.6	60.7	5.1	9
Total	61.2	68.2	7	11

*EAT* Ebola awareness tutorial, PHC primary healthcare

### Attitude toward EVD

Interestingly, health workers who feared EVD reduced significantly from 89 to 52%. Positive attitudes, e.g. avoiding handshakes with/touching Ebola patients, avoiding bush meat and avoiding to wash the corpses of diseased patients, were reinforced as indicated by increases between pre- and post-EAT scores from 83 to 92%, 57 to 64% and 67 to 79%, respectively (Table 5). Negative attitudes/perceptions such as allowing EVD patients to associate with family members, attributing EVD to spiritual attacks and believing EVD is treatable with antibiotics seemed to have been discouraged judging from the pre- and post-EAT scores.

**Table 5:** Attitudes and perceptions of frontline health workers to Ebola virus disease

Question	Responses	Pre-EAT no. (%)	Post-EAT no. (%)
How fearful are you of EVD?	Not fearful	13 (6)	2 (1)
	Undecided	10 (5)	96 (47)
	Fearful	180 (89)	105 (52)
Ebola can be prevented by avoiding handshakes and touching others during the epidemic	Agree	169 (83)	187 (92)
	Not sure	10 (5)	1 (0)
	Disagree	24 (12)	15 (7)
Ebola patients should be allowed to associate with family members	Agree	36 (18)	19 (9)
	Not sure	13 (6)	2 (1)
	Disagree	154 (76)	182 (90)
EVD is a spiritual attack	Agree	27 (13)	4 (2)
	Not sure	15 (7)	16 (8)
	Disagree	161 (79)	183 (90)
Antibiotics can cure EVD	Agree	42 (21)	18 (9)
	Not sure	30 (15)	26 (13)
	Disagree	131 (65)	159 (78)
EVD can be prevented by avoiding touching or eating bush meat	Agree	115 (57)	129 (64)
	Not sure	14 (7)	13 (6)
	Disagree	74 (36)	61 (30)
Washing the remains of deceased EVD patients causes no harm	Agree	46 (23)	32 (16)
	Not sure	21 (10)	11 (5)
	Disagree	136 (67)	160 (79)

*EAT* Ebola awareness tutorial, *EVD* Ebola virus disease

### Practice

Two questions indirectly assessed health workers’ practices with respect to EVD and infection control. This assessment was built on the premise that the Ebola tutorial may positively influence health workers’ response in the post-EAT survey. As expected, health workers responded more positively in favour of desirable clinical practices of frequent hand washing and disinfection of surfaces and equipment (Tables 6 and 7). Similarly, health workers responded more positively to using personal protective equipment for preventing the spread of EVD.

**Table 6:** Response to frequency of hand washing by health workers in the workplace

How often do you wash your hands in the workplace?	Pre-EAT no. (%)	Post-EAT no. (%)
Frequently	193 (95)	197 (97)
Sometimes	7 (3)	4 (2)
Rarely	3 (2)	2 (1)
Total	203 (100)	203 (100)

*EAT* Ebola awareness tutorial

**Table 7:** Response to frequency of washing surfaces and equipment

How often do you wash surfaces and equipment in the workplace?	Pre-EAT no. (%)	Post-EAT no. (%)
Frequently	191 (94)	196 (96.5)
Sometimes	5 (3)	4 (2)
Rarely	3 (1)	2 (1)
Not applicable	4 (2)	1 (0.5)
Total	203 (100)	203 (100)

*EAT* Ebola awareness tutorial

## Discussion

Following the Ebola epidemic in West Africa, a number of KAP studies were conducted among the general population and/or health workers in Nigeria [29, 30], Sierra Leone [31], Liberia [32] and Cameroon [33], with a view to using the findings to design strategies for preventing transmission and caring for people affected by Ebola. Similarly, new mHealth learning courses were developed to equip FHWs in affected countries with targeted, high-quality, up-to-date information as a pathway to building resilient health systems that can respond to the epidemic [8, 34]. However, the new mobile learning courses (e.g. mHero associated courses) implemented in Guinea, Liberia and Sierra Leone [8] are yet to be empirically evaluated to establish their effectiveness as health system-strengthening tools. To our knowledge, this study conducted in Nigeria is the first pre- and post-evaluation of a mHealth-enabled education intervention in West Africa that aimed to change the knowledge and attitude of FHWs to EVD.

Our findings mirror those of other studies in two ways. First, the revelation that most FHWs had heard of EVD before our evaluation is consistent with findings of KAP surveys conducted among the general population which showed that the majority of respondents in Lagos [29, 35] and all respondents in Sierra Leone [32] had foreknowledge of EVD before participating in respective surveys. Our finding that most FHWs were aware of EVD prior to the survey is unsurprising considering that the present pre- and post-intervention survey was conducted after Nigeria successfully quarantined all known cases of Ebola. Second, the finding from Ondo State that radio and TV were the dominant sources of EVD information is corroborated by studies in Lagos that identified television as the commonest source of information followed by radio [29], whereas radio was the dominant source of EVD information in Sierra Leone followed by television [32]. These findings together underline the importance of electronic media for disseminating information during such outbreaks.

The effectiveness of the FLEM application in changing FHWs’ knowledge of EVD may be ascribed to disseminating educational materials in real time combined with the convenience of unfettered access to online learning materials by FHWs over a stipulated time period. This underlines the potential benefits of electronic dissemination of educational information to enhance FHWs’ learning. It is noteworthy that improvements in average knowledge of EVD occurred across all cadres rather than in a given category of FHWs. It is, however, unclear whether the reduction in post-EAT scores relating to questions about the natural host of Ebola virus reflects existing uncertainty in the scientific community on this issue.

Besides modest improvements in knowledge, the post-EAT reinforcement of positive attitudes of avoiding handshakes, avoiding bush meat and avoiding risky burial practices during the Ebola epidemic is reassuring. Nevertheless, our findings should be interpreted with caution. Despite a growing recognition in the academic community that it is difficult to obtain reliable information on people’s knowledge and attitudes from KAP surveys [36, 37], we speculate that the modest positive changes in knowledge and attitudes reported by FHWs in Ondo State may be indirectly driven by a desire to use their learning to minimize the risk of contracting EVD in the context of a high case fatality rate and absence of treatment [4]. We, however, acknowledge that getting the general population to change age-long traditions of eating local delicacies or changing norms of social greeting can be a daunting endeavour. Also, maintaining close contact with the remains of deceased family members is commonplace in Nigeria, a practice that was implicated in the propagation of the EVD outbreak in West Africa [31]. During the EVD outbreak in Nigeria, a lot of misinformation was spread via social media about how drinking and/or bathing with salty water or eating bitter kola can prevent EVD [38]. Some faith healers even proclaimed their ability to cure EVD. Reports of surveys conducted in Lagos 2014 showed that 5% of health workers thought EVD was a spiritual problem while 25% of them believed that traditional medicine could cure EVD [35]. We believe delivering correct health information/educational materials quickly and efficiently to FHWs would have been invaluable in countering such false claims in Lagos [34]. Similarly, equipping FHWs with appropriate information is critical in countering misconceptions, e.g. about the role of traditional healers and funeral practices in relation to EVD.

In clinical settings, hand washing and disinfection of clinical areas and equipment remain the cornerstone of infection control. These practices are applicable to EVD prevention as the Ebola virus can be easily denatured by most standard disinfectants used in healthcare [39, 40]. In ideal conditions, the Ebola virus may remain infective on surfaces for up to 6 days [41, 42]. Getting health workers to appreciate the importance of hand washing and disinfection is vital especially when dealing with EVD outbreaks. However, in addition to education interventions discussed in the above paragraphs, we acknowledge that changing age-long practices requires addressing multiple factors ranging from socio-cultural to environmental, economic and structural factors that influence the logic behind people’s behaviour [37, 43].

## Conclusions

In conclusion, the FLEM mHealth tablet application appears to be effective for educating FHWs in the context of an epidemic. The modest changes in knowledge and attitudes recorded from the evaluation of this education intervention suggest that mHealth tutorial applications have potential for information dissemination and training of FHWs during EVD outbreaks. The user-friendly nature of this application and regular access to a limitless amount of educational materials, streamed in real time to FHWs, is a strong selling point of this application. Using mHealth tutorial applications as a means of bolstering the capability of health systems to manage disease conditions and improving health in LMICs should be explored further, especially given the increased global utilization of mobile technology and corresponding reduction in cost of mobile phone subscription.

### Limitations of the study

The study has four limitations. First, it is limited by the small sample of 282 FHWs, which reflects the fact that the mHealth intervention was implemented only in health facilities where CliniPAK wireless electronic education and health record systems were already provided in Ondo State. Second, we acknowledge that some of the improvement in knowledge and attitudes observed in this study may arise from parallel exposure of FHWs to, e.g. government-implemented mass media campaign about EVD during our research. Nevertheless, the evidence that the FLEM intervention is effective in improving KAP of FHWs sets the stage for expanding coverage and further testing of the intervention to other health facilities in the state. Third, the study is limited by a 2-month industrial strike during the study period that prevented some participants from completing the post-KAA after taking the pre-KAA survey and viewing the EAT. Fourth, we acknowledge that the accuracy of our findings could have been increased by supporting the KAP survey, e.g. with in-depth interviews and focus group discussions, to provide context to and clarify reasons behind some participant responses.

## Ethics approval

Health Research and Ethics Committee Number: G.8061/99

## Abbreviations

CHEW: community health extension worker
CHO: Community Health Officer
EVD: Ebola virus disease
FHWs: frontline health workers
FLEM: front line worker education and disease management
HRH: human resources for health
KAP: knowledge, attitude and practice
LGA: local government area
LMIC: low- and middle-income country
MCH: mother and child health
Post-EAT: post-Ebola awareness tutorial
Post-KAA: post-tutorial knowledge and attitude assessment
Pre-EAT: pre-Ebola awareness tutorial
Pre-KAA: pre-tutorial knowledge and attitude assessment
WHO: World Health Organization
